# Endocannabinoids Interact With the Dopaminergic System to Increase Sexual Motivation: Lessons From the Sexual Satiety Phenomenon

**DOI:** 10.3389/fnbeh.2019.00184

**Published:** 2019-08-14

**Authors:** Ana Canseco-Alba, Gabriela Rodríguez-Manzo

**Affiliations:** Departamento de Farmacobiología, Centro de Investigación y de Estudios Avanzados (Cinvestav-Sede Sur), Ciudad de México, México

**Keywords:** endocannabinoids (eCBs), CB1 receptors, D1-like/D2-like DA receptors, sexual motivation, sexual satiety, natural reward, mesolimbic circuit

## Abstract

In male rats, copulation to satiety induces a long-lasting sexual inhibitory state, considered to rely on a decreased sexual motivation. Dopaminergic transmission at the mesolimbic system plays a central role in the regulation of male sexual motivation. Endocannabinoids (eCBs) modulate the activity of the mesolimbic system and both dopamine (DA) and cannabinoid receptor activation reverses the sexual inhibition that characterizes sexually satiated rats. The eCB anandamide reverses sexual satiety when systemically administered or infused into the ventral tegmental area (VTA), the region where the activity of mesolimbic dopaminergic neurons is regulated. Thus, it could be thought that sexual motivation is diminished during the long-lasting sexual inhibition of sexually satiated rats and that eCBs reverse that inhibition through the modulation of the dopaminergic system. To test this hypothesis, we assessed the motivational state of sexually satiated male rats and determined if 2-arachidonoylglycerol (2-AG), the most abundant eCB and a full cannabinoid receptor agonist, also reversed the sexual inhibitory state. To establish the possible interaction between 2-AG and anandamide with the dopaminergic system for the reversal of sexual satiety, we analyzed the effects of the co-administration of each eCB and DA receptor agonists or antagonists. Results showed that 24-h after copulation to satiety, when the sexual inhibition is well established, the males’ sexual motivation is diminished as measured in the sexual incentive motivation test. 2-AG, similarly to anandamide, reverses sexual satiety through the activation of CB1 receptors and both eCBs interact with the dopaminergic system to reverse the sexual inhibitory state. 2-AG effects are mediated by the modulation of the D2-like DA receptor family, whereas anandamide’s effects are clearly mediated by the modulation of the D1-like DA receptor family and the activation of D2-like DA receptors. Present results evidence that a reduced sexual motivation underlies the sexual inhibitory state of sexually satiated rats and support the notion that eCBs reverse sexual satiety by modulating dopaminergic transmission, presumably at the mesolimbic system. Anandamide and 2-AG have a different interaction with D1-like and D2-like DA receptor families. Altogether present data endorse the association of the eCB system with the regulation of the motivational tone at the mesolimbic system.

## Introduction

Sexually experienced male rats allowed to copulate without restriction with a single female will ejaculate repeatedly until becoming sexually exhausted (Beach and Jordan, 1956; Rodríguez-Manzo and Fernández-Guasti, 1994). Copulation to satiety has as its main outcome the installation of a long- lasting sexual behavior inhibition (up to 72 h) that gradually fades away, requiring a 15-day period of sexual rest for exhausted males to completely recover their initial ejaculatory capacity (Rodríguez-Manzo et al., 2011). Twenty-four hours after copulation to satiety, when exposed to a new sexually receptive female, the majority of these animals (two-thirds of the population) does not show any sexual activity and the remaining third displays a single ejaculatory series after which males will not resume copulation (Rodríguez-Manzo and Fernández-Guasti, 1994).

Copulation is a highly rewarding behavior and the mesolimbic dopaminergic (MSL) system is involved in the control of its motivational component and reinforcing properties (Kelley and Berridge, 2002). The dopamine (DA) neurons of the MSL system, originating in the ventral tegmental area (VTA) of the midbrain, project to the nucleus accumbens (NAcc; Swanson, 1982; Ikemoto and Panksepp, 1999). DA has been suggested to be important for the assignment of the motivational value to rewarding behaviors (Berridge and Kringelbach, 2011) and motivation plays a central role in the maintenance of rewarding behaviors that are triggered by salient environmental stimuli, such as sexual behavior (Everitt, 1990). Copulation activates the MSL system increasing DA release at the NAcc (Mas et al., 1990; Pfaus et al., 1990; Wenkstern et al., 1993) and augmenting c-Fos protein expression in the DA neurons of the VTA (Balfour et al., 2004). During repeated copulation, DA levels at the NAcc remain elevated, indicating a continued activation of the MSL system (Fiorino et al., 1997).

The long-lasting sexual behavior inhibition that characterizes sexually exhausted male rats is considered to rely on a decreased sexual motivation (Guadarrama-Bazante and Rodríguez-Manzo, 2019), as their performance in a sexual motivation paradigm, immediately after reaching sexual satiety, is diminished (Ågmo et al., 2004). Besides, it has also been shown that interfering with the sexual motivation decline that follows copulation to exhaustion, by means of the Coolidge effect (renewal of sexual activity in satiated rats induced by changing the female partner), hinder the establishment of the long-lasting sexual behavior inhibition 24 h after copulation to satiety (Rodríguez-Manzo, 1999a). These data suggest that changes in the motivational component of copulatory behavior might play an important role in the sexual satiety phenomenon.

Interestingly, 24 h after copulation to exhaustion, once the sexual inhibitory state is established, changing the female partner has no effect on the sexual responsiveness of the satiated rats (Rodríguez-Manzo, 1999a). Though, the established sexual inhibition can be reversed by a number of pharmacological agents (a 5-HT1A receptor agonist, an α2-adrenoceptor antagonist, μ and δ opioid antagonists, among others), acting at different neurotransmitter systems, which seem to directly or indirectly interact with the dopaminergic system (Rodríguez-Manzo and Fernández-Guasti, 1995; Rodríguez-Manzo, 1999b). In addition, DA receptor agonists, systemically administered or infused into the NAcc, also reverse the sexual inhibition of satiated rats (Guadarrama-Bazante et al., 2014; Guadarrama-Bazante and Rodríguez-Manzo, 2019). Together, these data suggest that DA transmission plays a central role in the reversal of sexual satiety.

Endocannabinoids (eCBs) are retrograde transmitters, of which anandamide (AEA) and 2-arachidonoylglycerol (2-AG) are the best characterized (Di Marzo et al., 1998). Unlike classical neurotransmitters, eCBs are synthesized and released on demand, during periods of high neural activity (Freund et al., 2003). At the MSL system, eCBs are released from the DA cell bodies in the VTA and from the medium spiny neurons in the NAcc (Lupica and Riegel, 2005). Once in the synaptic cleft, they retrogradely activate CB1 cannabinoid receptors, located on GABAergic and glutamatergic axon terminals in each of these brain regions, thereby inhibiting neurotransmitter release (Alger, 2002; Wilson and Nicoll, 2002). Through the modulation of MSL system’s activity, eCBs regulate rewarding behaviors (Lupica et al., 2004; Gardner, 2005). Sexual behavior is rewarding and eCBs are involved in its control (Gorzalka et al., 2008), playing a complex role in its expression (for review, see Rodríguez-Manzo and Canseco-Alba, 2015).

In sexually satiated male rats, low doses of AEA reverse the sexual inhibition that characterizes sexual satiety (Canseco-Alba and Rodríguez-Manzo, 2014), an effect mimicked by its direct infusion into the VTA (Canseco-Alba and Rodríguez-Manzo, 2016).

Based on these data, it could be thought that sexual motivation is diminished during the long-lasting sexual inhibitory period that characterizes sexually exhausted rats and that eCBs reverse that inhibition through the modulation of the dopaminergic system. To test this hypothesis, in this work we first assessed the motivational state of sexually exhausted male rats 24 h after copulation to satiety, by means of a sexual incentive motivation test. We then determined if 2-AG, the most abundant eCB in the brain, also reversed sexual satiety through the activation of CB1 receptors. Finally, we analyzed the possibility of an interaction between AEA or 2-AG and the dopaminergic system for the reversal of sexual satiety, determining the possible participation of each of the two DA receptor families in this effect.

## Materials and Methods

### Animals

**Figure 1:**
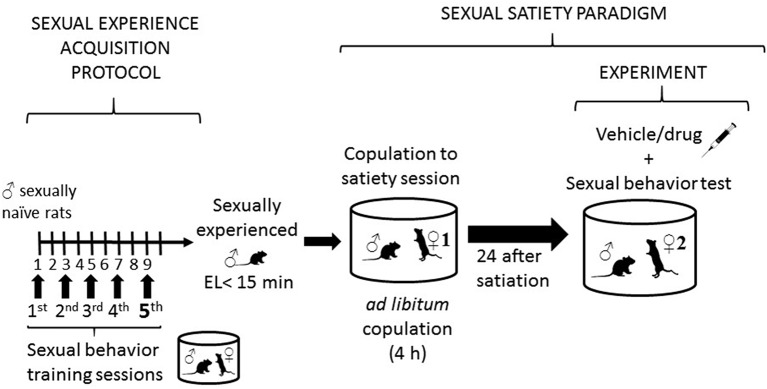
Description of sexual behavior protocols. The left side of the figure describes the protocol followed to render sexually experienced male rats, consisting in subjecting sexually naïve rats to five independent sexual behavior sessions, run every other day. At the end of this process males showing ejaculation latencies (EL) shorter than 15 min are considered sexually experienced and selected for the study. The right side of the figure describes the sexual satiety paradigm, involving sexually experienced male rats that copulate *ad libitum* with a single sexually receptive female (♀1) until reaching the sexual satiety criterion (around 4 h). Twenty-four hours later these males receive the pharmacological treatment or its vehicle and are subjected to a sexual behavior test with a new sexually receptive female (♀2).

Sexually experienced adult male Wistar rats (250–300 g b. wt.) were used in this study. Animals were housed, eight per cage, under inverted light/dark cycle conditions (12 h light: 12 h dark, lights on at 22:00 h), at 22°C, and with free access to food and water. For the selection of sexually experienced males, rats were subjected to five independent sexual behavior tests, and those males showing ejaculation latencies (EL) shorter than 15 min, in at least three of these tests, were considered sexually experienced (see Figure 1). Receptive female Wistar rats served as sexual stimuli. Sexual receptivity was induced in intact females by the sequential s.c. injection of estradiol benzoate (12 μg/rat) followed 24 h later by progesterone (6.0 mg/rat). Our institutional Internal Committee for the Care and Use of Laboratory Animals (Comité Institucional para el Cuidado y Uso de Animales de Laboratorio, CICUAL) approved all experimental procedures (Protocol 0230-16), which followed the regulations established in the Mexican Official Norm for the use and care of laboratory animals NOM-062-ZOO-1999.

### Sexual Exhaustion Paradigm

Sexual behavior observations were conducted in a room under dim red light, during the dark phase of the cycle. Male rats were introduced into polycarbonate cylindrical arenas (62 cm diameter, 52 cm height), with the floor covered with fine sawdust, and a 5-min adaptation period was allowed to the males before introducing a receptive female. The males copulated with a single receptive female during 4 h, without restriction. Previous data from our laboratory have shown that this period is sufficient for all animals to reach the sexual exhaustion criterion, i.e., 90 min from the last ejaculation without attaining another ejaculation. At the end of the sexual exhaustion session, the animals were returned to their home cages. Twenty-four hours later, the same animals were subjected to a sexual behavior test with a new sexually receptive female, after receiving the pharmacological treatments or the vehicle (see Figure 1).

In this last test, we recorded the percentage of males displaying sexual behavior, i.e., mount, intromission, ejaculation and copulation resumption after ejaculation. Since these animals are sexually inhibited, the display of each of these sexual responses indicates a facilitation of sexual behavior expression. When the proportion of satiated animals capable of resuming copulation after a first ejaculation, during the 24 h test, is significantly increased in response to a pharmacological treatment, it is considered that sexual satiety was reversed. In those animals ejaculating, we recorded the following specific sexual parameters: intromission latency (IL, time from the introduction of the female to the appearance of the first intromission); mount and intromission number displayed prior to ejaculation (M and I); EL (time from the first intromission until ejaculation) and post-ejaculatory interval (PEI, time from ejaculation to the first intromission of the next copulatory series). These specific parameters are regularly used to evaluate the sexual performance of sexually experienced male rats.

### Locomotor Activity

In order to discard non-specific effects of the drug treatments that could have interfered with sexual behavior execution, the animals’ spontaneous locomotor activity was recorded immediately after the sexual behavior tests that followed drug treatments. To this purpose, male rats were placed into an acrylic box (33 × 44 × 20 cm), with the floor divided into 12 squares (11 × 11 cm for each quadrant), and the number of crossings from one quadrant to another during a 5-min period was recorded. The cage was carefully cleaned between tests.

### Sexual Incentive Motivation Test

The sexual incentive motivation test was conducted in a room under dim red light following the method described by Ågmo (2003). This is a non-conditioned test measuring the sexual incentive motivation induced in male rats by a sexually receptive female as opposed to the social incentive motivation induced by another male rat. The apparatus consists of a solid plastic elliptic open field arena (85 × 50 × 40 cm) that has two diagonally opposed windows (one in each long wall extreme), separated from the central arena by wire mesh. Each of these windows communicates with a removable incentive animal cage (20 × 10 × 15 cm), separated from the arena by the wire mesh in which the incentive animals, i.e., a sexually receptive female or a sexually experienced male are placed. In front of each window, a rectangular zone in front of each incentive animal cage (measuring 30 × 20 cm) is designated as the incentive zone. Between tests, the female and male cages are semi-randomly changed from one position to another and the apparatus cleaned to eliminate odor traces from other animals.

Prior to the experimental session, the male subjects are habituated to the arena for three consecutive days in the absence of incentive animals and allowed to freely explore it for 10 min. On the test day, the incentive animals are introduced into their cages, the experimental male is then placed into the center of the arena where it can hear, see and smell the inaccessible incentive animals and its behavior is videotaped during 10 min, in the absence of the experimenter. An observer, blind to the experimental groups, analyzed video recordings. The cumulative time spent by the experimental subjects in the respective incentive zones is considered as indicative of the incentive motivation generated by each animal (male or female).

### Drugs

All drugs were purchased from Sigma-Aldrich Chem. Company (St. Louis, MO, USA). Arachidonoylethanolamide (anandamide, AEA) and 2-AG were dissolved in a vehicle composed by a mixture of ethanol (2%), Tween80 (2%) and saline solution (96%). AM251 was dissolved in a vehicle composed by a mixture of DMSO (1 drop), Tween (2%) and saline solution (98%). Haloperidol was dissolved in distilled water adding three drops of ascorbic acid (0.01%). Apomorphine, quinpirole, SKF38399, SCH23390 and raclopride were dissolved in saline solution. All drugs were i.p. injected in a volume of 1 ml/kg. All the CB1 ligands (AEA, 2-AG and AM251) were administered 5 min before subjecting the animals to the sexual behavior tests. The DA receptor ligands had different latencies, which are specified for each drug in the experimental design. Estradiol benzoate and progesterone were dissolved in sesame oil and s.c. injected to the females as described above, under the animals’ heading.

### Statistical Analyses

Comparison of the proportions of sexually exhausted rats exhibiting the different sexual behavior responses, i.e., mount, intromission, ejaculation and copulation resumption after ejaculation, was conducted by means of the Fisher *F*-test. The distinct sexual behavior parameters of sexually experienced males in the dose-response curves, as well as the locomotor activity data were compared by means of the Kruskal–Wallis ANOVA followed by Dunn’s test when pertinent. The differences in the time spent by male rats in the different incentive zones were established by means of the Mann-Whitney *U* test. All statistical analyses were performed with the Sigma Plot program (version 12.0).

### Experimental Design

#### Experiment 1: Incentive Sexual Motivation of Sexually Exhausted Male Rats

Two independent groups of sexually experienced male rats (*n* = 12 each) were used. One group was directly tested for incentive motivation and served as the control group. The experimental group was first subjected to the sexual exhaustion paradigm and 24 h after copulation to satiety, tested for incentive motivation.

#### Experiment 2: Effects of 2-AG on Sexual Behavior Expression of Sexually Experienced and Sexually Exhausted Male Rats

A dose-response (D-R) curve of the effects of 2-AG (0.03–3.0 mg/kg) in sexually experienced rats was run to establish the effects of this eCB on copulation of sexually active animals. To establish the effects of 2-AG in sexually satiated rats, six independent groups of sexually experienced males (*n* = 8 each) were subjected to the sexual exhaustion paradigm and 24 h later, injected with different doses of 2-AG (0.03–3.0 mg/kg) or its vehicle and their sexual activity recorded. An additional group of sexually exhausted rats was employed to establish if 2-AG effects were mediated by CB1 receptors. In this case, the CB1 receptor antagonist AM251 (0.1 mg/kg) was injected to the satiated male rats immediately before the administration of an effective 2-AG dose (0.3 mg/kg) and after 5 min the sexual behavior test was run. The AM251 dose was chosen from a previously reported D-R curve (Canseco-Alba and Rodríguez-Manzo, 2014).

#### Experiment 3: Interaction of the eCBs AEA and 2-AG With the Dopaminergic System in Sexually Exhausted Male Rats

Four independent groups of sexually exhausted males (*n* = 8 each) were used to establish the effects of the unspecific DA receptor antagonist haloperidol (125 μg/kg, −30 min) on the reversal of sexual exhaustion induced by an effective dose of AEA (0.3 mg/kg) or 2-AG (0.3 mg/kg). The AEA dose was chosen from the D-R curve of AEA effects on sexually satiated males previously reported (Canseco-Alba and Rodríguez-Manzo, 2014). The haloperidol dose was chosen from a published D-R curve run in sexually satiated animals (Rodríguez-Manzo, 1999b) and was injected 30 min prior to either eCB; the control group received the combination of vehicles.

The possible interaction of the unspecific DA receptor agonist, apomorphine with the eCBs AEA and 2-AG was determined by the co-administration of the DA agonist and each of the eCBs, at doses that were subthreshold for reversing sexual satiety. To this aim, four additional independent groups of satiated males (*n* = 8 each) were used; one receiving the combination of vehicles, another receiving the sub-effective dose of apomorphine (10 μg/kg, −15 min) and two for the combinations of apomorphine with either AEA or 2-AG, at sub-effective doses (0.03 mg/kg each). Apomorphine’s ineffective dose was chosen from a pilot study with sexually experienced male rats. The data of this pilot study are shown in Table 1.

**Table 1:** Specific sexual behavior parameters of the first copulatory series of sexually experienced male rats treated with specific doses of dopamine (DA) receptor agonists.

Treatment	*n*	IL	M	I	EL	PEI
Vehicle (saline)	8	0.91 ± 0.17	3	8	8.07 ± 0.76	5.68 ± 0.34
Apomorphine 10 μg/kg	8	0.68 ± 0.14	2	6.5	6.04 ± 0.79	5.78 ± 0.54
SKF-38399 0.1 mg/kg	8	0.80 ± 0.18	5	10	9.77 ± 1.0	6.03 ± 0.47

*IL, intromission latency; M, number of mounts; I, number of intromissions; EL, ejaculation latency; PEI, postejaculatory interval. Temporal measures are expressed in minutes (min) as mean ± SEM and M&I as median number*.

#### Experiment 4: Participation of D1-Like DA Receptors in the eCB-Induced Reversal of Sexual Satiety

Two independent groups of sexually satiated rats (*n* = 8 each) were used to establish the effects of the combined treatment of an effective dose of AEA or 2-AG (0.3 mg/kg each) with a dose of the D1-like receptor antagonist SCH23390 that lacked effects *per se* (0.1 mg/kg, −30 min). A dose-response curve of SCH23390 was run in sexually experienced male rats to identify the dose not modifying sexual behavior *per se* (Figure 6).

**Figure 2:**
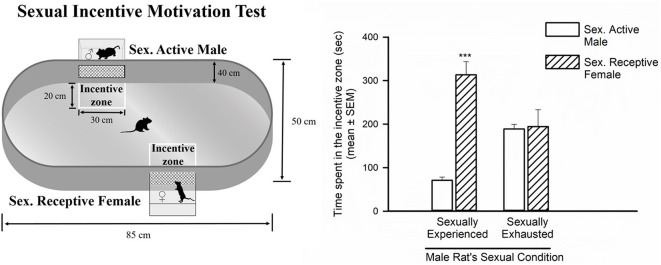
Sexual incentive motivation is measured as the time spent by the male rat in the incentive zones of the sexually receptive female (dashed bars) or the male (empty bars), in control sexually experienced (*n* = 12) and in sexually satiated male rats (*n* = 12). Data in the graph are expressed in seconds (s) as mean ± SEM. A statistically significant difference was found in the sexually experienced males in the time spent in each of the incentive zones. Mann-Whitney *U* test, ***P < 0.001. The features of the apparatus used to evaluate sexual incentive motivation are depicted on the left side of the figure.

**Figure 3:**
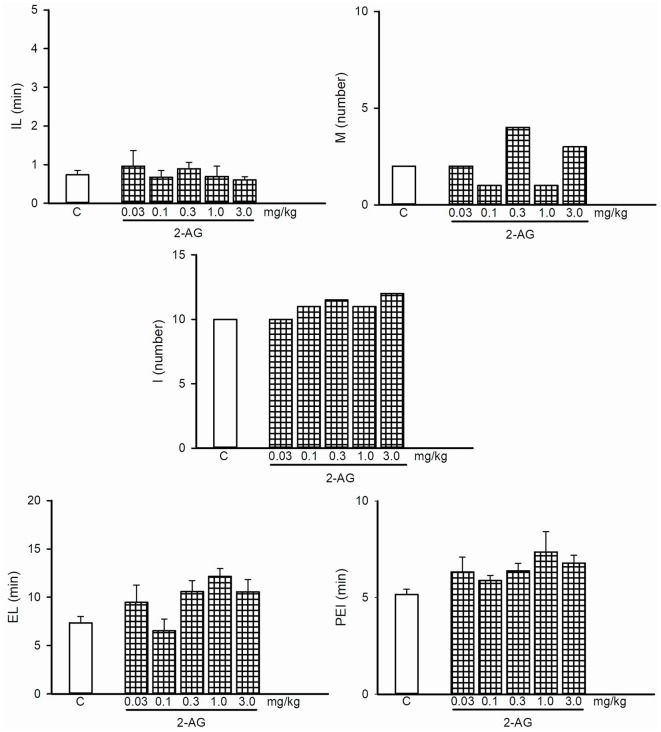
Dose-response curve of the effects of different doses of 2-arachidonoylglycerol (2-AG; 0.03–3.0 mg/kg, *n* = 8 each) or vehicle (C) on the specific sexual behavior parameters of sexually experienced male rats. IL, intromission latency; M, number of mounts; I, number of intromissions; EL, ejaculation latency; PEI, postejaculatory interval. Latencies are expressed in minutes, as mean ± SEM, and numbers as medians.

**Figure 4:**
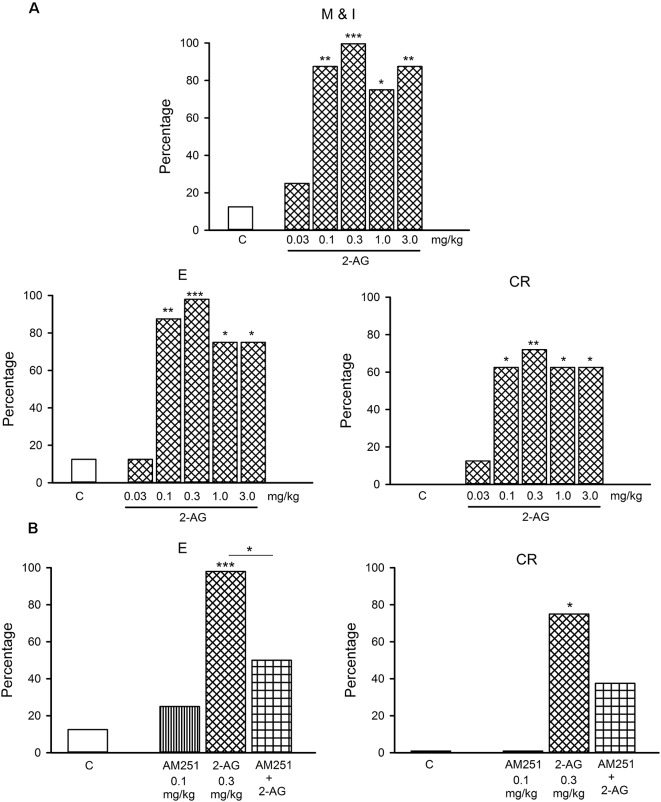
**(A)** Dose-response curve of the effects of different doses of 2-AG (0.03–3.0 mg/kg) or vehicle (C) on the percentage of sexually satiated rats that exhibited the different sexual behavior responses: mount and intromission (M and I), ejaculation (E) and copulation resumption after ejaculation (CR). Fisher *F* test, *P < 0.05, **P < 0.01, ***P < 0.001 vs. C. Panel **(B)** shows that the CB1 receptor antagonist, AM251 (0.1 mg/kg), blocks the reversal of sexual exhaustion induced by 0.3 mg/kg 2-AG, i.e., the increase in the percentage of satiated rats ejaculating (E) and resuming copulation after ejaculation (CR). Fisher *F*-test *P < 0.05; ***P < 0.001; *n* = 8 for each group. Asterisks over bars indicate statistical significance vs. the control group; other comparisons are indicated.

**Figure 5:**
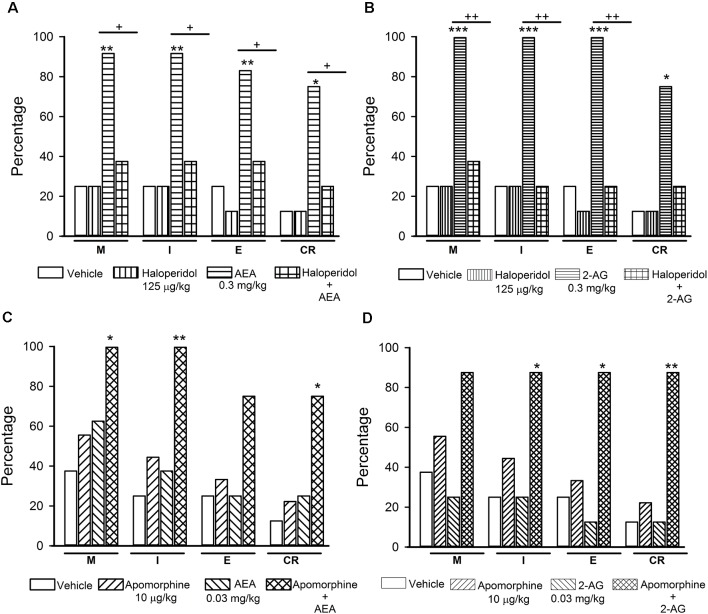
Interaction of the endocannabinoids (eCBs) AEA and 2-AG with dopamine (DA) transmission in the reversal of sexual satiety. The upper graphs show the percentages of sexually exhausted rats that are able to mount (M), intromit (I), ejaculate (E) and resume copulation after ejaculation (CR) following vehicle (1 ml/kg), Haloperidol (125 μg/kg), AEA (0.3 mg/kg) or 2-AG (0.3 mg/kg) and the combined treatment of Haloperidol with AEA (panel **A**) or 2-AG (panel **B**). The lower graphs depict the effects of vehicle (1 ml/kg), Apomorphine (10 μg/kg) and their combined treatment with AEA (0.03 mg/kg; panel **C**) or 2-AG (0.03 mg/kg; panel **D**) on the percentages of satiated rats showing M, I, E and CR. Fisher *F*-test, *P < 0.05, **P < 0.01, ***P < 0.001, ^+^*P* < 0.05, ^++^*P* < 0.01; *n* = 8 for each group. Asterisks over bars indicate statistical significance vs. the control group; crosses show significance for other indicated comparisons.

**Figure 6:**
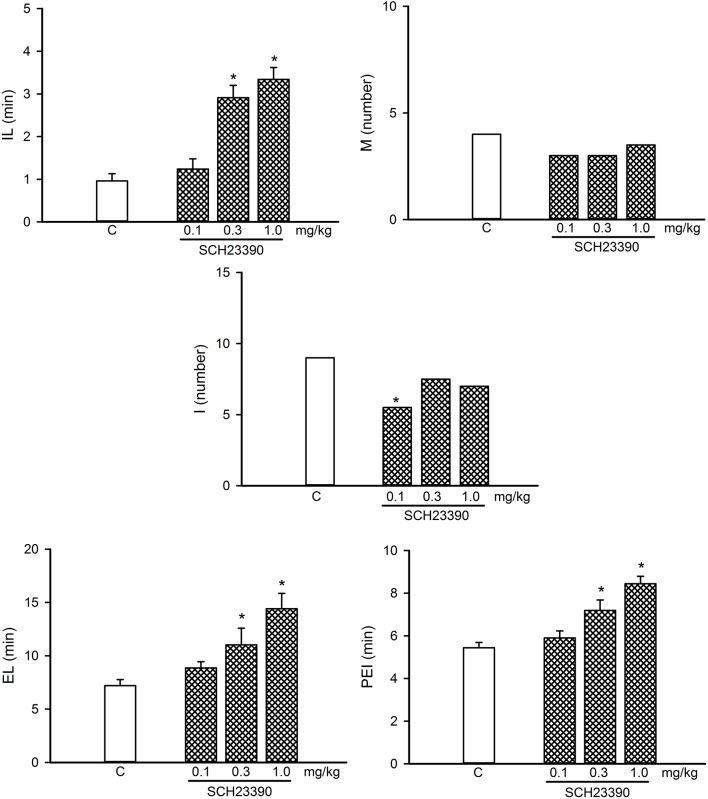
Dose-response curve of the effects of different doses of the D1-like receptor antagonist, SCH23390 (0.1–1.0 mg/kg, *n* = 8 each), on sexual behavior of sexually experienced male rats. IL, intromission latency; M, number of mounts; I, number of intromissions; EL, ejaculation latency; PEI, postejaculatory interval; C, control. Latencies are expressed in minutes, as mean ± SEM, and numbers as medians. Kruskal–Wallis ANOVA followed by Dunn’s test, *P < 0.05 vs. control.

Two additional groups of sexually exhausted males (*n* = 8 each) were employed to establish the effects of the combination of previously determined suboptimal doses of AEA or 2-AG (0.03 mg/kg each) with a sub-effective dose of the D1-like receptor agonist SKF38399 (0.1 mg/kg, −30 min), which was determined in a pilot study with sexually experienced male rats. The data of this pilot study are included in Table 1.

#### Experiment 5: Participation of D2-Like DA Receptors in the eCB-Induced Reversal of Sexual Satiety

Two independent groups of sexually satiated rats (*n* = 8 each) were used to establish the effects of the combined treatment of an effective dose of AEA or 2-AG (0.3 mg/kg each) with a dose of the D2-like receptor antagonist raclopride that lacked effects *per se* (0.03 mg/kg, −20 min). A dose–response curve of raclopride was run in sexually experienced male rats to identify the dose not modifying sexual behavior (Figure 8).

**Figure 7:**
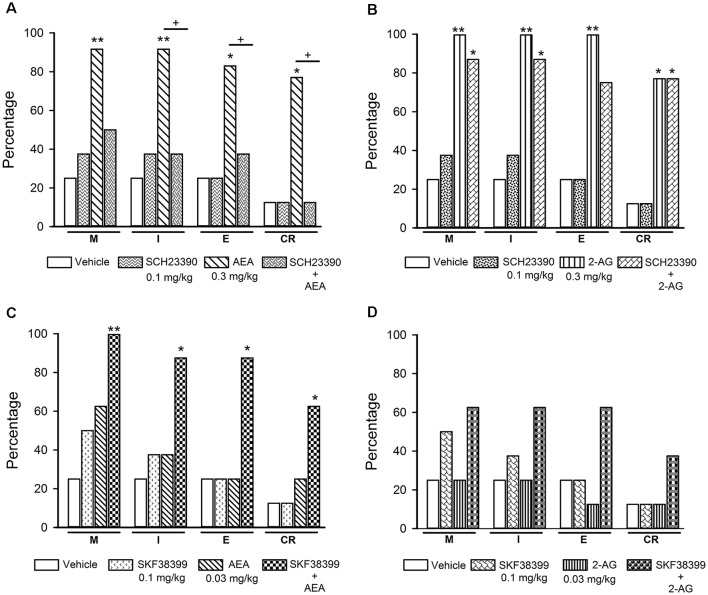
Effects of the combined treatment of eCBs and D1-like receptor ligands on sexual behavior of sexually exhausted male rats. The upper graphs show the percentages of sexually satiated rats that are able to mount (M), intromit (I), ejaculate (E) and resume copulation after ejaculation (CR) following vehicle (1 ml/kg), the D1-like receptor antagonist SCH23390 (0.1 mg/kg), AEA (0.3 mg/kg) or 2-AG (0.3 mg/kg) and the combined treatment of SCH23390 with AEA (panel **A**) or with 2-AG (panel **B**). The lower graphs depict the effects of vehicle (1 ml/kg), the D1-like receptor agonist SKF38399 (0.1 mg/kg) and their combined treatment with AEA (0.03 mg/kg; panel **C**) or 2-AG (0.03 mg/kg; panel **D**) on the percentages of satiated rats showing M, I, E and CR. Fisher *F* test, *P < 0.05, **P < 0.01, ^+^*P* < 0.05; *n* = 8 for each group. Asterisks over bars indicate statistical significance vs. the control group; crosses show significance for other indicated comparisons.

**Figure 8:**
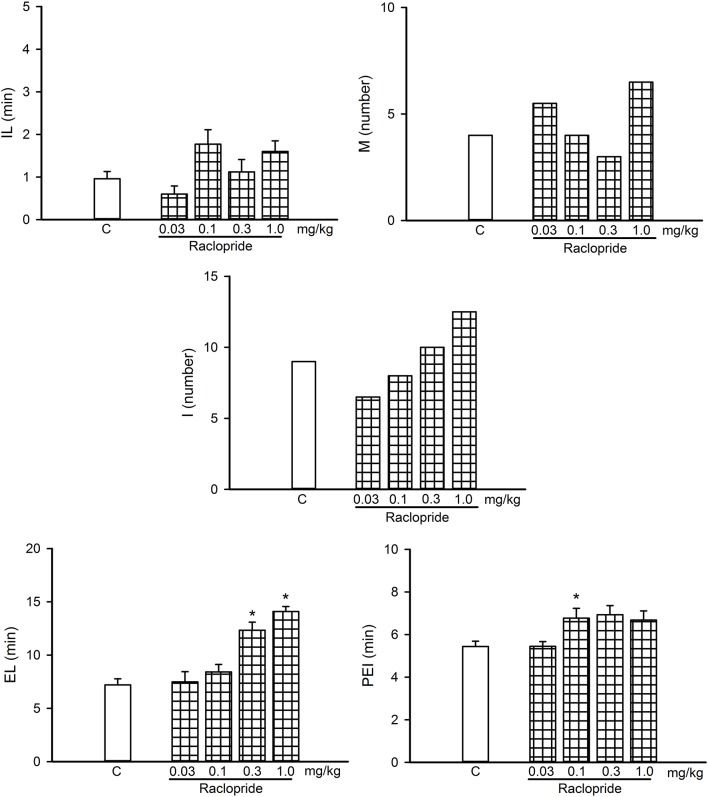
Dose-response curve of the effects of different doses of the D2-like receptor antagonist Raclopride (0.03–1.0 mg/kg, *n* = 8 each) on sexual behavior of sexually experienced male rats. IL, intromission latency; M, number of mounts; I, number of intromissions; EL, ejaculation latency; PEI, postejaculatory interval; C, control. Latencies are expressed in minutes as mean ± SEM and numbers as medians. Kruskal–Wallis ANOVA followed by Dunn’s test, *P < 0.05 vs. control.

Finally, another two groups of sexually exhausted males (*n* = 8 each) were employed to establish the effects of the combination of suboptimal doses of AEA or 2-AG (0.03 mg/kg each) with a suboptimal dose of the D2-like receptor agonist quinpirole (0.03 mg/kg, −15 min) chosen from a previously reported D-R curve in sexually satiated rats (Guadarrama-Bazante et al., 2014).

## Results

### Sexual Incentive Motivation of Sexually Exhausted Male Rats

In the sexual incentive motivation test it was found that sexually experienced male rats spent significantly more time in the incentive zone of the sexually receptive female as compared to the time spent in the male’s incentive zone (Mann-Whitney *U* test, *U* = 2, *P* < 0.001). In contrast, in the sexually exhausted males, tested 24 h after copulation to satiety, there was no difference between the time spent by the males in the incentive zones of the sexually receptive female and the sexually active male (Mann-Whitney *U* test, *U* = 82.5, *P* = 0.93; Figure 2).

### Effects of 2-AG on Sexual Behavior of Sexually Experienced and Sexually Exhausted Male Rats

Figure 3 depicts the dose-response curve of the effects of different doses of 2-AG on the sexual behavior of sexually experienced male rats. It can be observed that none of the sexual parameters were statistically significantly modified by any 2-AG dose. The percentage of sexually exhausted male rats showing mounts and intromissions (M&I), ejaculating (E) and resuming copulation after ejaculation (CR), 24 h after copulation to satiety, in response to different doses of the eCB 2-AG, are shown in Figure 4. As it can be seen in panel **A**, the majority of the tested doses (0.1–3.0 mg/kg) significantly increased the proportion of satiated rats attaining ejaculation [seven out of eight (87.5%; Fisher *F* test, *P* = 0.01) for 0.1 mg/kg; eight out of eight (100%; Fisher *F* test, *P* = 0.001) for 0.3 mg/kg and six out of eight (75%; Fisher *F* test, *P* < 0.05) for both the 1.0 and 3.0 mg/kg doses] and resuming copulation thereafter [five out of eight (62.5%; Fisher *F* test, *P* < 0.05) for 0.1 mg/kg; six out of eight (75%; Fisher *F* test, *P* < 0.01) for 0.3 mg/kg; and five out of eight (62.5%; Fisher *F* test, *P* < 0.05) for the 1.0 and 3.0 mg/kg doses], while the lowest dose tested (0.03 mg/kg) failed to increase these proportions. Thus, 2-AG doses between 0.1 and 3.0 mg/kg reversed sexual satiety.

Figure 4B depicts the action of the CB1 receptor antagonist, AM251, at a dose that lacks effects *per se* (0.1 mg/kg), on the increase in the percentages of sexually exhausted rats ejaculating and resuming copulation after ejaculation induced by 0.3 mg/kg 2-AG. It can be observed that the 2-AG-induced reversal of sexual satiety was canceled, indicating that this effect is mediated by CB1 receptors.

### Interaction of the eCBs AEA and 2-AG With the Dopaminergic System in Sexually Exhausted Male Rats

Figure 5 shows the effects of the combined injection of the unspecific DA receptor antagonist haloperidol with AEA or 2-AG (panels **A** and **B**, respectively) and those of the combined injection of apomorphine, a non-specific DA receptor agonist, with AEA or 2-AG (panels **C** and **D**, respectively) in sexually exhausted male rats. Haloperidol injection (125 μg/kg) *per se* did not induce mating behavior in sexually satiated rats, while a dose of 0.3 mg/kg AEA (panel **A**) or 2-AG (panel **B**), statistically significantly increased the proportions of satiated rats ejaculating (Fisher *F* test, *P* < 0.01 for AEA; *P* < 0.001 for 2-AG) and resuming copulation after ejaculation (Fisher *F* test, *P* < 0.05 for both eCBs), as compared to vehicle-treated satiated males. Pre-treatment with haloperidol canceled both, AEA- and 2-AG-induced increases in these percentages.

Apomorphine, at the dose of 10 μg/kg, lacked effects on copulation of satiated rats, as did AEA (panel **C**) and 2-AG (panel **D**) at the dose of 0.03 mg/kg. However, the combined administration of AEA with apomorphine increased the proportion of sexually exhausted males copulating. These increases were statistically significant for M (Fisher *F* test, *P* < 0.05), for I (Fisher *F* test, *P* < 0.01) and for CR (Fisher *F* test, *P < 0.05*). The combined treatment of apomorphine with 2-AG statistically significantly increased the proportion of satiated rats showing I (Fisher *F* test, *P* < 0.05), E (Fisher *F* test, *P* < 0.05) and CR (Fisher *F* test, *P* = 0.01). Thus, sub-effective doses of apomorphine and each of the eCBs synergized to reverse sexual satiety.

### Participation of D1-Like DA Receptors in the eCB-Induced Reversal of Sexual Satiety

A dose-response curve of the effects of the D1-like receptor antagonist SCH23390 on the sexual behavior of sexually experienced male rats is shown in Figure 6. SCH23390 significantly increased the temporal parameters in these animals at the 0.3 and 1.0 mg/kg doses (Kruskal–Wallis ANOVA *H*_(3)_ = 24.66, *P* < 0.001; Dunn’s Test, *P* < 0.05 for IL), (Kruskal–Wallis ANOVA *H*_(3)_ = 16.36, *P* < 0.001; Dunn’s Test, *P* < 0.05 for EL; Kruskal–Wallis ANOVA *H*_(3)_ = 20.33, *P* < 0.001; Dunn’s Test, *P* < 0.05 for PEI). The lowest SCH23390 dose (0.1 mg/kg) reduced the I number (Kruskal–Wallis ANOVA *H*_(3)_ = 8.18, *P* < 0.042; Dunn’s Test, *P* < 0.05), considered a sexual facilitative outcome, and lacked effects on any other parameter; therefore, this dose was selected for the combined treatments.

Figure 7 depicts the effects of the combined injection of the D1-like receptor antagonist SCH23390 with effective doses of AEA (panel **A**) or 2-AG (panel **B**), as well as the effects of the combination of sub-effective doses of the D1-like receptor agonist SKF-38399 with sub-effective doses of AEA (panel **C**) or 2-AG (panel **D**), on sexual behavior expression of sexually satiated rats. It can be seen that the sole administration of 0.1 mg/kg SCH23390 lacked effects in sexually satiated rats, however, it canceled the increase in the percentage of satiated rats showing sexual behavior induced by an effective dose of AEA (panel **A**). By contrast, this same SCH23390 dose did not block the actions of the 2-AG effective dose on copulation of sexually satiated rats (panel **B**). This figure also shows that combination of sub-effective doses of the D1-like receptor agonist with AEA synergized to significantly increase the proportion of satiated rats showing each of the sexual behavior responses (Fisher *F* test, *P* < 0.01 for M; *P* < 0.05 for I, E and CR; panel **C**), whereas its combination with a sub-effective dose of 2-AG failed to significantly increase these proportions (panel **D**).

### Participation of D2-Like DA Receptors in the eCB-Induced Reversal of Sexual Satiety

Figure 8 shows a dose-response curve of the effects of the D2-like receptor antagonist raclopride on the sexual behavior of sexually experienced male rats. Raclopride had sexual effects at doses from 0.1 to 1.0 mg/kg, increasing the EL (Kruskal–Wallis ANOVA, *H*_(3)_ = 26.36, *P* < 0.001; Dunn’s test, *P* < 0.05) at the two higher doses and the PEI at the lower dose (Kruskal–Wallis ANOVA, *H*_(3)_ = 20.33, *P* < 0.001; Dunn’s test, *P* < 0.05). We selected the lowest raclopride dose tested (0.03 mg/kg) for the combined treatments, as it lacked sexual effects.

**Figure 9:**
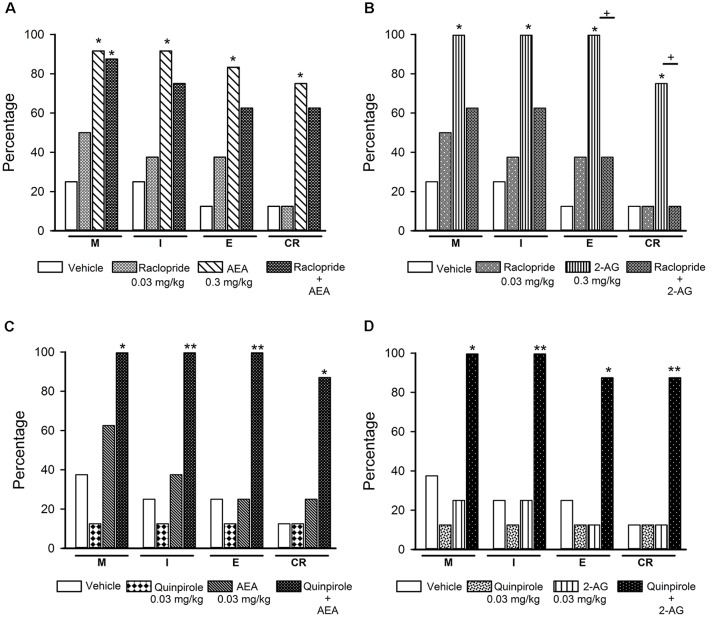
Effects of the combined treatment of eCBs and D2-like receptor ligands on sexual behavior of sexually exhausted male rats. The upper graphs show the percentages of sexually satiated rats that are able to mount (M), intromit (I), ejaculate (E) and resume copulation after ejaculation (CR) following vehicle (1 ml/kg), the D2-like receptor antagonist Raclopride (0.03 mg/kg), AEA (0.3 mg/kg) or 2-AG (0.3 mg/kg) and the combined treatment of Raclopride with AEA (panel **A**) or 2-AG (panel **B**). The lower graphs depict the effects of vehicle (1 ml/kg), the D2-like receptor agonist Quinpirole (0.03 mg/kg) and their combined treatment with AEA (0.03 mg/kg; panel **C**) or 2-AG (0.03 mg/kg; panel **D**) on the percentages of satiated rats showing M, I, E and CR. Fisher *F* test, *P < 0.05, **P < 0.01, ^+^*P* < 0.05; *n* = 8 for each group. Asterisks over bars indicate statistical significance vs. the control group; crosses show significance for other indicated comparisons.

The effects of the combined injection of raclopride with effective doses of AEA or 2-AG on sexual behavior expression of sexually exhausted male rats are shown in Figure 9 (panels **A** and **B**, respectively), as well as the effects of the combination of sub-effective doses of the D2-like receptor agonist quinpirole with sub-effective doses of AEA (panel **C**) or 2-AG (panel **D**). It can be observed that 0.03 mg/kg raclopride, *per se*, lacked effects on copulation of satiated rats. When combined with an effective dose of AEA, raclopride did not block the increases induced by this eCB (panel **A**), however, it canceled the 2-AG-induced increases in the proportion of satiated animals capable of displaying the different sexual behavior responses (panel **B**). Combination of sub-effective doses of the D2-like receptor agonist quinpirole and the eCBs, AEA or 2-AG, synergized to significantly increase the proportion of satiated rats showing each of the sexual behavior responses. The combination of quinpirole with AEA promoted M, I and E display in all animals (Fisher *F* test, *P* = 0.02 for M; *P* = 0.007 for I and E) and seven out of eight animals resumed copulation thereafter (Fisher *F* test, *P* = 0.01). Combined treatment of quinpirole with 2-AG induced M and I behavior in all animals (Fisher *F* test, *P* = 0.026 for M and *P* = 0.007 for I), while seven out of eight animals ejaculated (Fisher *F* test, *P* = 0.041) and resumed copulation after ejaculation (Fisher *F* test, *P* = 0.01; panels **C** and **D**, respectively).

The specific sexual behavior parameters of the satiated animals in which treatments reversed satiety are shown in Table 2.

**Table 2:** Specific sexual behavior parameters of those sexually satiated male rats achieving ejaculation in response to drug treatment and of a group of sexually experienced male rats as a reference.

Treatment	*n*	IL	M	I	EL	PEI
2-AG 0.3 mg/kg	6/8	13.55 ± 2.45	6	9	9.29 ± 2.93	15.67 ± 2.92
Apomorphine 10 μg/kg + AEA 0.03 mg/kg	7/8	3.91 ± 0.68	3	7	5.62 ± 1.05	16.02 ± 2.70
Apomorphine 10 μg/kg + 2-AG 0.03 mg/kg	7/8	3.01 ± 1.24	6	10	10.07 ± 3.02	16.41 ± 1.58
SKF-38399 0.1 mg/kg + AEA 0.03 mg/kg	6/8	3.62 ± 0.56	3	8	8.57 ± 1.80	20.32 ± 3.03
Quinpirole 0.03 mg/kg + AEA 0.03 mg/kg	8/8	4.22 ± 2.43	1	3	9.17 ± 2.43	23.92 ± 3.18
Quinpirole 0.03 mg/kg + 2-AG 0.03 mg/kg	7/8	13.18 ± 6.32	1	7	6.37 ± 0.84	38.05 ± 8.23
Sexually experienced	8/8	2.22 ± 0.28	0.5	6.5	5.97 ± 0.64	5.50 ± 0.61

*IL, intromission latency; M, number of mounts; I, number of intromissions; EL, ejaculation latency; PEI, postejaculatory interval. Temporal measures are expressed in minutes (min) as mean ± SEM and M and I as median number*.

None of the pharmacological treatments significantly affected the spontaneous ambulatory behavior of sexually satiated male rats. These data are presented in Table 3. Table 1 includes the data of the pilot studies showing the doses of apomorphine and SKF-38399 that lacked effects *per se* on the sexual behavior of sexually experienced male rats which were selected for experiments involving combined treatments of sub-effective drug doses.

**Table 3:** Effect of the different drug treatments on spontaneous locomotor activity of sexually exhausted male rats.

Drug	Dose	Number of counts/5 min mean ± SEM
2-AG Veh	0	44.25 ± 3.41
2-AG	0.03 mg/kg	47.50 ± 2.86
	0.1 mg/kg	47.25 ± 6.05
	0.3 mg/kg	51.75 ± 6.18
	1.0 mg/kg	46.75 ± 5.76
	3.0 mg/kg	48.88 ± 6.08
AM251 Veh + 2AG Veh	0	42.00 ± 3.13
AM251+2-AG	0.1 mg/kg + 0.1 mg/kg	49.50 ± 1.60
Haloperidol	125 μg/kg	46.71 ± 2.19
Hal Veh + AEA/2-AG Veh	0	41.10 ± 2.31
Haloperidol + AEA	125 μg/kg + 0.3 mg/kg	38.12 ± 1.97
Haloperidol + 2-AG	125 μg/kg + 0.3 mg/kg	41.03 ± 2.47
saline + AEA/2-AG Veh	0	35.87 ± 2.09
Apomorphine	10 μg/kg	36.25 ± 1.81
Apomorphine+ AEA	10 μg/kg + 0.03 mg/kg	38.25 ± 1.08
Apomorphine+ 2-AG	10 μg/kg + 0.03 mg/kg	35.75 ± 3.29
SCH23390	0.1 mg/kg	37.00 ± 2.61
SCH23390 + AEA	0.1 mg/kg +0.3 mg/kg	32.62 ± 1.48
SCH23390 + 2-AG	0.1 mg/kg + 0.3 mg/kg	50.38 ± 3.77
Raclopride	0.03 mg/kg	30.37 ± 1.58
Raclopride + AEA	0.03 mg/kg + 0.3 mg/kg	32.87 ± 2.60
Raclopride + 2-AG	0.03 mg/kg + 0.3 mg/kg	47.01 ± 3.20
SKF38399	0.1 mg/kg	38.28 ± 3.47
SKF38399 + AEA	0.1 mg/kg + 0.03 mg/kg	33.14 ± 2.25
SKF38399+ 2-AG	0.1 mg/kg + 0.03 mg/kg	44.57 ± 4.65
Quinpirole	0.03 mg/kg	56.11 ± 7.85
Quinpirole + AEA	0.03 mg/kg + 0.03 mg/kg	47.86 ± 1.90
Quinpirole + 2-AG	0.03 mg/kg + 0.03 mg/kg	50.25 ± 3.83

*Kruskal–Wallis ANOVAs, non-significant*.

## Discussion

The main findings of the present series of experiments can be summarized as follows: (a) sexually exhausted male rats exhibit a reduced sexual motivation 24 h after copulation to satiety, when the sexual inhibitory period is well established; (b) low doses of the eCB 2-AG reverse sexual satiety through a CB1 receptor-dependent mechanism, but do not modify copulatory behavior of sexually experienced male rats; (c) the eCBs AEA and 2-AG interact with the dopaminergic system to induce sexual behavior expression in sexually exhausted male rats; (d) D2-like, but not D1-like DA receptors, participate in the 2-AG-induced sexual satiety reversal; and (e) AEA-induced satiety reversal is mediated by D1-like DA receptors. Notwithstanding, D2-like receptor agonists also synergize with AEA to induce sexual activity in sexually exhausted males.

The sexual incentive motivation test revealed that sexually exhausted male rats do not show the preference for a sexually receptive female exhibited by sexually experienced animals, thereby confirming that during the sexual inhibitory period that characterizes sexual satiety, male rats have a reduced sexual motivation. As mentioned in the introduction section, previous data implied that sexual motivation might play a role in the sexual satiety phenomenon (Rodríguez-Manzo, 1999a; Guadarrama-Bazante and Rodríguez-Manzo, 2019), including a report on a diminished sexual motivation measured in male rats immediately after reaching sexual satiety (Ågmo et al., 2004). However, the sexual motivational state of the satiated animals during the long-lasting sexual inhibitory period (i.e., 24–72 h later) had not been directly assessed. This assessment is important, because 24 h after copulation to satiety, not only the characteristic sexual inhibitory state is well established, but also other possible confounding factors are absent. For instance, the fatigue due to intense copulation is no longer present, since the animals rested overnight. Males had free access to water and food when returning to their home-cages after copulation to satiety, eliminating hunger and thirst as factors playing a role in their lack of interaction with the receptive female rat. Finally, during the sexual incentive motivation test, the sexually satiated rats are exposed to a new sexually receptive female, eliminating the possible habituation to the sexual partner as another factor involved in the absence of preference. Under these conditions, we believe that the lack of interest for the sexually receptive female exhibited by sexually satiated males 24 h after copulation to satiety reflects an actual decrease in sexual motivation, thus validating the notion that this factor is involved in the long-lasting sexual inhibitory state that characterizes the sexual satiety phenomenon.

eCB signaling in the brain has been found to regulate the motivation for natural rewards (Parsons and Hurd, 2015). Reinforcing this notion, previous data from our group showed that the eCB AEA reversed sexual satiety after its direct infusion into the VTA (Canseco-Alba and Rodríguez-Manzo, 2016). This result supports the idea that eCBs’ actions at the MSL system might modify the sexual motivational tone of sexually satiated rats. AEA and 2-AG are the best-characterized eCBs and both activate CB1 receptors; however, AEA binds with moderate affinity and is a partial agonist at CB1 receptors, whereas 2-AG binds with low affinity but exhibits full efficacy at these receptors (Hillard, 2000). Besides, 2-AG is more abundant than AEA in the brain (Stella et al., 1997; Nomura et al., 2008) and is considered as the key eCB released on demand by VTA DA neurons to modulate its own activity (Melis et al., 2004; Tanimura et al., 2010). Therefore, it was crucial to determine if 2-AG was also capable of reversing the sexual inhibitory state of sexually exhausted rats. Results showed that similar to AEA’s effects (Canseco-Alba and Rodríguez-Manzo, 2014), low doses of 2-AG reversed sexual satiety through the activation of CB1 receptors. However, 2-AG was capable of inducing copulation in satiated rats within a broader dose range than AEA (0.1–3.0 mg/kg vs. 0.1–0.3 mg/kg, respectively), which is compatible with the higher efficacy of the former at CB1 receptors. Interestingly, 2-AG induced the display of sexual behavior in sexually satiated rats but did not modify the sexual performance of sexually experienced animals. A possible explanation for this differential action could be based on the proposal that the net effect of CB1 receptor-mediated actions at the MSL system depend on the level of baseline activity of midbrain DA neurons, such that enhancing DA neuronal firing may have a larger effect when baseline frequency is low compared to when neurons are burst firing (Covey et al., 2017). Following this idea, when sexually experienced rats are exposed to sexually receptive females as well as during copulation, DA neurons fire in the bursting mode rendering a phasic DA release in the NAcc (Robinson et al., 2002). Although, eCBs are capable of removing the tonic inhibition exerted by GABAergic inputs onto midbrain dopaminergic neurons to promote the phasic mode of DA release (Oleson et al., 2012), in the sexually experienced males this effect is also elicited by the rewarding stimulus (Grace, 1991; Grace et al., 2007), i.e., the presence and interaction with the sexually receptive female, which could account for the lack of 2-AG sexual facilitative effects in these animals. By contrast, in sexually satiated rats the inhibition of their sexual responsiveness suggests that DA neuron baseline activity is low, explaining the ability of 2-AG to produce sexual behavior facilitative effects in animals with this sexual condition.

A central finding of the present work is that both AEA and 2-AG interact with the dopaminergic system to reverse sexual satiety. Sub-optimal doses of each of these eCBs synergized with a dose of apomorphine that was subthreshold for reversing sexual satiety, whereas the reversal of the sexual inhibitory state, induced by effective doses of AEA or 2-AG, was canceled by the DA receptor antagonist haloperidol. These results contribute to strengthening the notion of a relationship between eCB and DA signaling in the facilitation of reward-motivated behaviors (Oleson and Cheer, 2012; Wenzel and Cheer, 2018). They also suggest that the deficient motivational tone of sexually exhausted rats might be increased by eCBs’ actions, through the modulation of DA activity, enabling sexually satiated males to respond with sexual activity to the rewarding stimulus, represented by the receptive female.

It has been proposed that the dopaminergic system might be the common final pathway for the pharmacological reversal of sexual satiety. This idea emerged from the fact that several drugs, acting at different neurotransmitter systems, are capable of reversing sexual satiety by interacting, directly or indirectly, with the dopaminergic system (Rodríguez-Manzo and Fernández-Guasti, 1995; Rodríguez-Manzo, 1999b; Hull and Rodríguez-Manzo, 2017). Present results are in line with this proposal ascribing to the MSL system a central position in the dopaminergic-mediated regulation of male rat sexual behavior expression.

Midbrain DA neurons are involved in the signaling of reward-related stimuli by changing their firing pattern (Grace, 1991; Grace et al., 2007). The basal activity of these neurons involves low-frequency firing resulting in a dopaminergic tone capable of activating high-affinity D2-like DA receptors in the NAcc. Upon the presentation of a rewarding stimulus, this firing pattern changes to a high-frequency burst firing that is accompanied by an increase in NAcc DA release, which activates low-affinity D1-like DA receptors (Grace et al., 2007; Dreyer et al., 2010). On these bases, establishing the DA receptor family/families involved in the 2-AG/DA and AEA/DA interactions to induce sexual behavior display in sexually satiated rats appeared relevant. Interestingly, our data showed that there was a differential interaction between each of these eCBs and the two DA receptor families. 2-AG synergized with D2-like, but not with D1-like DA receptor agonists, to induce sexual satiety reversal; an effect that was completely prevented by the D2-like receptor antagonist raclopride. In contrast, AEA synergized with both D1- and D2-like DA receptor agonists to reverse the sexual inhibition, but only the D1-like DA receptor antagonist SCH23390 was able to block the AEA-induced sexual satiety reversal.

It has been documented that 2-AG and AEA, in spite of activating the same cannabinoid receptors and signal transduction pathways (Janero et al., 2009), do not always play the same physiological role, acting sometimes in concert and sometimes not (Di Marzo and Cristino, 2008; Luchicchi and Pistis, 2012). It is important to recall that in the present work eCBs were exogenously administered and, therefore, could activate CB1 receptors in different brain regions. Within the MSL system, CB1 receptors are expressed both in the NAcc (Pickel et al., 2006) and in the VTA (Herkenham et al., 1991); therefore, systemically administered eCBs might have reversed satiety by acting at each of these brain regions, where they behave as retrograde messengers suppressing presynaptic glutamate and GABA release (Lupica and Riegel, 2005). However, substantial evidence indicates that exogenously administered cannabinoids increase DA release in rat NAcc (Chen et al., 1990; Gardner and Vorel, 1998; Gessa et al., 1998) and excite midbrain DA neurons in the VTA (French et al., 1997). These data suggest that the actions of exogenously administered eCBs in the present work would be exerted at the VTA; a proposal supported by the finding that intra-VTA infusion of AEA reverses sexual satiety (Canseco-Alba and Rodríguez-Manzo, 2016).

In relation to DA receptors, D1-like and D2-like receptors within the MSL system are mainly expressed in the NAcc, segregated in different populations of medium spiny neurons, which constitute 95% of the cells in this brain region (Yang et al., 2018). According to present data, the 2-AG/DA interaction for the reversal of sexual satiety clearly involves only the activation of D2-like receptors, probably from the NAcc, since in the VTA these receptors are somatodendritic autoreceptors that regulate the firing rate of DA neurons and DA release in terminal fields. D2 autoreceptor activation reduces DA release at the NAcc and also limit somatodendritic DA release in the VTA (Rice and Patel, 2015). In support of the notion that the 2-AG/DA interaction takes place in the NAcc is the finding that direct infusion of the selective D2-like DA receptor agonist, quinpirole, into this brain region reverses the sexual inhibition of satiated rats (Guadarrama-Bazante and Rodríguez-Manzo, 2019).

Results of this work also suggest that AEA’s interaction with DA transmission in sexually satiated rats is essentially, although not exclusively, mediated by D1-like DA receptor activation. This conclusion derives from the fact that D1-like DA receptor blockade with the antagonist SCH23390 canceled AEA-induced reversal of sexual satiety and a sub-effective dose of the D1-like receptor agonist SKF33939 synergized with a dose of AEA, that was subthreshold for reversing sexual satiety, to promote sexual behavior display. In line with this finding, our group observed that systemically administered DA receptor agonists reverse sexual satiety through the activation of D1-like DA receptors (Guadarrama-Bazante et al., 2014).

AEA also synergized with D2-like DA receptor activation to reverse sexual satiety, but reversal of sexual satiety induced by an effective dose of AEA, was not canceled by the D2-like receptor antagonist raclopride. These results evidence that AEA effects are mediated by the modulation of D1-like DA receptors, though the independent activation of D2-like DA receptors with quinpirole could synergize with the AEA-mediated activation of D1-like DA receptors to reverse satiety. Interestingly, activation of D2-like DA receptors in the NAcc increases the extracellular levels of AEA in this brain region (Giuffrida et al., 1999). In fact, DA exerts modulatory effects on both AEA and 2-AG content in the NAcc (Patel et al., 2003), where medium spiny neurons synthesize and release these eCBs in response to DA stimulation. Remarkably, the NAcc’s content of AEA and 2-AG is differentially modulated by the activation of D1-like and D2-like DA receptors, respectively (Patel et al., 2003). These data further support the relationships described in the present work, between AEA and D1-like DA receptors and between 2-AG and D2-like DA receptors. Thus, a possible contribution to the reversal of sexual satiety of changes in NAcc’s content of these two eCBs, resulting from the activation of the two DA receptor families, cannot be discarded.

In spite of the differential interaction found for each eCB with the DA receptor families, it has been considered that the simultaneous activation of D1-like and D2-like DA receptors in the NAcc is required for the processing of reward relevant information (Ikemoto et al., 1997). Moreover, it has been reported that only the combination of D1-like and D2-like receptor agonists is able to enhance NAcc core cell firing *in vitro*—an effect in which the participation of eCBs is required—, while the independent activation of each of these receptor families does not reproduce this result (Seif et al., 2011). Remarkably, we found that the direct infusion of the non-selective DA receptor agonist, apomorphine, into the NAcc induced a full sexual satiety reversal, not obtained with any other pharmacological treatment so far tested in sexually satiated rats. In this case, the most effective apomorphine dose induced sexual behavior to ejaculation and copulation resumption after ejaculation in every sexually satiated animal. Besides, the copulatory performance of these males was as efficient as that of sexually experienced males, an outcome not commonly seen with other treatments reversing satiety (Guadarrama-Bazante and Rodríguez-Manzo, 2019). This result reinforces the notion that the regulation of the rewarding properties and motivational tone at the NAcc is mediated by the cooperative actions of the two DA receptor families.

Based on the data here presented, it could be proposed that repeated activation of the MSL system by intense copulation during sexual satiety development, induces a diminished sexual motivational tone, responsible for their characteristic long-lasting sexual behavior inhibition. AEA and 2-AG seem to reverse this sexual inhibitory state by modulating dopaminergic transmission, presumably at the MSL system. Specific experiments further analyzing the role played by eCBs in the regulation of DA-mediated motivation for natural rewards at the MSL system are warranted.

The motivational component of male rat sexual behavior and the initiation of the copulatory behavioral pattern (Blackburn et al., 1992) have not only been linked to mesolimbic DA, but also to DA transmission at the hypothalamic medial preoptic area (mPOA; Pfaus and Phillips, 1991; Hull et al., 1999). Indeed, DA levels increase simultaneously in the NAcc and mPOA in response to the presence of an inaccessible sexually receptive female (Blackburn et al., 1992; Hull et al., 1999). However, present and previous data from our lab do not suggest a participation of this brain region in the eCB/DA-mediated increase in sexual motivation that leads to the reversal of sexual satiety. To the extent of our knowledge, eCBs have not been associated to the control of mPOA DA neuron activity and, infusion of DA receptor agonists into this brain region does not reverse sexual satiety, except for a specific low dose of the D2-like receptor agonist quinpirole (Guadarrama-Bazante and Rodríguez-Manzo, 2019). However, the systemic administration of several quinpirole doses failed to reverse the sexual inhibition of satiated rats (Guadarrama-Bazante et al., 2014). Notwithstanding, the indirect participation of the mPOA, a brain region that is crucial for male sexual behavior expression in all vertebrate species (Hull et al., 1999), through its efferent projections, which have been considered essential for the initiation of the copulatory pattern (Everitt, 1990), cannot be discarded. Actually, some of these projections target specifically the VTA (Simerly and Swanson, 1988; Stolzenberg and Numan, 2011; Zahm et al., 2011) and might influence the activity of mesolimbic DA neurons favoring an increase in sexual motivation of sexually satiated rats.

The present work uses sexual satiety as an animal model to provide new evidence showing that eCBs modulate the MSL system with an impact on sexual motivation. This model offers the opportunity to explore the nature of adaptive inhibitory mechanisms (Bancroft, 1999) controlling the expression of an innate behavior normally triggered by specific incentive stimuli, i.e., copulation in the presence of an accessible sexually receptive female, which appears to involve changes in the male’s motivational tone. This research also suggests that CB1 receptor activation might have therapeutic potential to positively influence sexual arousal and desire, which deficiency underlies several human sexual disorders, the hypoactive sexual desire disorder (American Psychiatric Association, 2013) among them; this possibility should be explored.

Like all studies, the present work has its limitations. Since all drug treatments in this study were systemically administered, the involvement of brain structures outside the mesolimbic system as well as peripheral effects of eCBs cannot be discarded. The participation of the MSL system in the eCB-mediated actions here described has to be confirmed, however, this study is a good starting point motivating more specific experiments directed to identify the brain loci within the mesolimbic system where the described eCB/DA interactions occur.

## Data Availability

The datasets generated for this study are available on request to the corresponding author.

## Ethics Statement

### Animal Subjects

The animal study was reviewed and approved by Cinvestav Internal Committee for the Care and Use of Laboratory Animals (Comité Institucional para el Cuidado y Uso de Animales de Laboratorio, CICUAL) following the regulations established in the Mexican Official Norm for the use and care of laboratory animals NOM-062-ZOO-1999; Protocol 0230-16.

## Author Contributions

AC-A and GR-M designed the experiments, performed statistics, interpreted data, drafted the manuscript, wrote the final manuscript and analyzed behavioral data. AC-A performed some behavioral experiments. GR-M conceptualized the study. Both authors contributed to the scientific discussions and approved the final manuscript.

## Conflict of Interest Statement

The authors declare that the research was conducted in the absence of any commercial or financial relationships that could be construed as a potential conflict of interest.

## References

[B1] Ågmo A. (2003). Unconditioned sexual incentive motivation in the male Norway Rat (*Rattus norvegicus*). J. Comp. Psychol. 117, 3–14. 10.1037/0735-7036.117.1.3 12735358

[B2] Ågmo A. Turi A. L. Ellingsen E. Kaspersen H. (2004). Preclinical models of sexual desire: conceptual and behavioural analysis. Pharmacol. Biochem. Behav. 78, 379–404. 10.1016/j.pbb.2004.04.013 15251248

[B3] Alger B. E. (2002). Retrograde signaling in the regulation of synaptic transmission: focus on endocannabinoids. Prog. Neurobiol. 68, 247–286. 10.1016/s0301-0082(02)00080-1 12498988

[B4] American Psychiatric Association (2013). Diagnostic and Statistical Manual of Mental Disorders. 5th Edn. Washington, DC: American Psychiatric Association.

[B5] Balfour M. E. Yu L. Coolen L. M. (2004). Sexual behavior and sex-associated environmental cues activate the mesolimbic system in male rats. Neuropsychopharmacology 29, 718–730. 10.1038/sj.npp.1300350 14694350

[B6] Bancroft J. (1999). Central inhibition of sexual response in the male: a theoretical perspective. Neurosci. Biobehav. Rev. 23, 763–784. 10.1016/s0149-7634(99)00019-6 10541055

[B7] Beach F. Jordan L. (1956). Sexual exhaustion and recovery in the male rat. Q. J. Exp. Psychol. 8, 121–133. 10.1080/17470215608416811

[B8] Berridge K. C. Kringelbach M. L. (2011). Building a neuroscience of pleasure and well-being. Psychol. Well Being 1, 1–3. 10.1186/2211-1522-1-3 22328976 PMC3274778

[B9] Blackburn J. R. Pfaus J. G. Phillips A. G. (1992). Dopamine functions in appetitive and defensive behaviours. Prog. Neurobiol. 39, 247–279. 10.1016/0301-0082(92)90018-a 1502338

[B10] Canseco-Alba A. Rodríguez-Manzo G. (2014). Low anandamide doses facilitate male rat sexual behaviour through the activation of CB1 receptors. Psychopharmacology 231, 4071–4080. 10.1007/s00213-014-3547-9 24671517

[B11] Canseco-Alba A. Rodríguez-Manzo G. (2016). Intra-VTA anandamide infusion produces dose-based biphasic effects on male rat sexual behavior expression. Pharmacol. Biochem. Behav. 150–151, 182–189. 10.1016/j.pbb.2016.11.004 27856203

[B12] Chen J. P. Paredes W. Li J. Smith D. Lowinson J. Gardner E. L. (1990). Delta 9-tetrahydrocannabinol produces naloxone-blockable enhancement of presynaptic basal dopamine efflux in nucleus accumbens of conscious, freely-moving rats as measured by intracerebral microdialysis. Psychopharmacology 102, 156–162. 10.1007/bf02245916 2177204

[B13] Covey D. P. Mateo Y. Sulzer D. Cheer J. F. Lovinger D. M. (2017). Endocannabinoid modulation of dopamine neurotransmission. Neuropharmacology 124, 52–61. 10.1016/j.neuropharm.2017.04.033 28450060 PMC5608040

[B14] Di Marzo V. Cristino L. (2008). Why endocannabinoids are not all alike. Nat. Neurosci. 11, 124–126. 10.1038/nn0208-124 18227793

[B15] Di Marzo V. Melck D. Bisogno T. De Petrocellis L. (1998). Endocannabinoids: endogenous cannabinoid receptor ligands with neuromodulatory action. Trends Neurosci. 21, 521–528. 10.1016/s0166-2236(98)01283-1 9881850

[B16] Dreyer J. K. Herrik K. F. Berg R. W. Hounsgaard J. D. (2010). Influence of phasic and tonic dopamine release on receptor activation. J. Neurosci. 30, 14273–14283. 10.1523/JNEUROSCI.1894-10.2010 20962248 PMC6634758

[B17] Everitt B. J. (1990). Sexual motivation: a neural and behavioural analysis of the mechanisms underlying appetitive and copulatory responses of male rats. Neurosci. Biobehav. Rev. 14, 217–232. 10.1016/s0149-7634(05)80222-2 2190121

[B18] Fiorino D. F. Coury A. Phillips A. G. (1997). Dynamic changes in nucleus accumbens dopamine efflux during the Coolidge effect in male rats. J. Neurosci. 17, 4849–4855. 10.1523/JNEUROSCI.17-12-04849.1997 9169543 PMC6573325

[B19] French E. D. Dillon K. Wu X. (1997). Cannabinoids excite dopamine neurons in the ventral tegmentum and substantia nigra. Neuroreport 8, 649–652. 10.1097/00001756-199702100-00014 9106740

[B20] Freund T. F. Katona I. Piomelli D. (2003). Role of endogenous cannabinoids in synaptic signaling. Physiol. Rev. 83, 1017–1066. 10.1152/physrev.00004.2003 12843414

[B22] Gardner E. (2005). Endocannabinoid signaling system and brain reward: emphasis on dopamine. Pharmacol. Biochem. Behav. 81, 263–284. 10.1016/j.pbb.2005.01.032 15936806

[B21] Gardner E. L. Vorel S. R. (1998). Cannabinoid transmission and reward-related events. Neurobiol. Dis. 6, 502–533. 10.1006/nbdi.1998.0219 9974181

[B23] Gessa G. L. Melis M. Muntoni A. L. Diana M. (1998). Cannabinoids activate mesolimbic dopamine neurons by an action on cannabinoid CB1 receptors. Eur. J. Pharmacol. 341, 39–44. 10.1016/s0014-2999(97)01442-8 9489854

[B24] Giuffrida A. Parsons L. H. Kerr T. M. Rodríguez de Fonseca F. Navarro M. Piomelli D. (1999). Dopamine activation of endogenous cannabinoid signaling in dorsal striatum. Nat. Neurosci. 2, 358–363. 10.1038/7268 10204543

[B25] Gorzalka B. Morrish A. Hill G. (2008). Endocannabinoid modulation of male rat sexual behavior. Psychopharmacology 198, 479–486. 10.1007/s00213-007-0901-1 17694389

[B26] Grace A. A. (1991). Phasic versus tonic dopamine release and the modulation of dopamine system responsivity: a hypothesis for the etiology of schizophrenia. Neuroscience 41, 1–24. 10.1016/0306-4522(91)90196-u 1676137

[B27] Grace A. A. Floresco S. B. Goto Y. Lodge D. J. (2007). Regulation of firing of dopaminergic neurons and control of goal-directed behaviors. Trends Neurosci. 30, 220–227. 10.1016/j.tins.2007.03.003 17400299

[B29] Guadarrama-Bazante I. L. Canseco-Alba A. Rodríguez-Manzo G. (2014). Dopamine receptors play distinct roles in sexual behavior expression of rats with a different sexual motivational tone. Behav. Pharmacol. 25, 684–694. 10.1097/fbp.0000000000000086 25171081

[B28] Guadarrama-Bazante I. L. Rodríguez-Manzo G. (2019). Nucleus accumbens dopamine increases sexual motivation in sexually satiated male rats. Psychopharmacology 236, 1303–1312. 10.1007/s00213-018-5142-y 30536080

[B30] Herkenham M. Lynn A. B. Johnson M. R. Melvin L. S. de Costa B. R. Rice K. C. (1991). Characterization and localization of cannabinoid receptors in rat brain: a quantitative *in vitro* autoradiographic study. J. Neurosci. 11, 563–583. 10.1523/JNEUROSCI.11-02-00563.1991 1992016 PMC6575215

[B31] Hillard C. J. (2000). Biochemistry and pharmacology of the endocannabinoids arachidonylethanolamide and 2-arachidonylglycerol. Prostaglandins Other Lipid Mediat. 61, 3–18. 10.1016/s0090-6980(00)00051-4 10785538

[B32] Hull E. M. Lorrain D. S. Du J. Matuszewich L. Lumley L. A. Putnam S. K.. (1999). Hormone-neurotransmitter interactions in the control of sexual behavior. Behav. Brain Res. 105, 105–116. 10.1016/s0166-4328(99)00086-8 10553694

[B33] Hull E. M. Rodríguez-Manzo G. (2017). “Male sexual behavior,” in Hormones, Brain, and Behavior, 3rd Edn. eds Pfaff D. W. Joëls M. (Oxford: Academic Press), 1–57.

[B34] Ikemoto S. Panksepp J. (1999). The role of nucleus accumbens dopamine in motivated behavior: a unifying interpretation with special reference to reward-seeking. Brain Res. Rev. 31, 6–41. 10.1016/s0165-0173(99)00023-5 10611493

[B35] Ikemoto S. Glazier B. S. Murphy J. M. McBride W. J. (1997). Role of dopamine D1 and D2 receptors in the nucleus accumbens in mediating reward. J. Neurosci. 17, 8580–8587. 10.1523/JNEUROSCI.17-21-08580.1997 9334429 PMC6573749

[B36] Janero D. R. Vadivel S. K. Makriyannis A. (2009). Pharmacotherapeutic modulation of the endocannabinoid signalling system in psychiatric disorders: drug-discovery strategies. Int. Rev. Psychiatry 21, 122–133. 10.1080/09540260902782778 19367506 PMC5531754

[B37] Kelley A. E. Berridge K. C. (2002). The neuroscience of natural rewards: relevance to addictive drugs. J. Neurosci. 22, 3306–3311. 10.1523/JNEUROSCI.22-09-03306.2002 11978804 PMC6758373

[B38] Luchicchi A. Pistis M. (2012). Anandamide and 2-arachidonoylglycerol: pharmacological properties, functional features, and emerging specificities of the two major endocannabinoids. Mol. Neurobiol. 46, 374–392. 10.1007/s12035-012-8299-0 22801993

[B39] Lupica C. R. Riegel A. C. Hoffman A. F. (2004). Marijuana and cannabinoid regulation of brain reward circuits. Br. J. Pharmacol. 143, 227–234. 10.1038/sj.bjp.0705931 15313883 PMC1575338

[B40] Lupica C. R. Riegel A. C. (2005). Endocannabinoid release from midbrain dopamine neurons: a potential substrate for cannabinoid receptor antagonist treatment of addiction. Neuropharmacology 48, 1105–1116. 10.1016/j.neuropharm.2005.03.016 15878779

[B41] Mas M. Gonzalez-Mora J. L. Louilot A. Solé C. Guadalupe T. (1990). Increased dopamine release in the nucleus accumbens of copulating male rats as evidenced by *in vivo* voltammetry. Neurosci. Lett. 110, 303–308. 10.1016/0304-3940(90)90864-6 2325902

[B42] Melis M. Pistis M. Perra S. Muntoni A. L. Pillolla G. Gessa G. L. (2004). Endocannabinoids mediate presynaptic inhibition of glutamatergic transmission in rat ventral tegmental area dopamine neurons through activation of CB1 receptors. J. Neurosci. 24, 53–62. 10.1523/jneurosci.4503-03.2004 14715937 PMC6729571

[B43] Nomura D. K. Hudak C. S. Ward A. M. Burston J. J. Issa R. S. Fisher K. J.. (2008). Monoacylglycerol lipase regulates 2-arachidonoylglycerol action and arachidonic acid levels. Bioorg. Med. Chem. Lett. 18, 5875–5878. 10.1016/j.bmcl.2008.08.007 18752948 PMC2593629

[B45] Oleson E. B. Beckert M. V. Morra J. T. Lansink C. S. Cachope R. Abdullah R. A.. (2012). Endocannabinoids shape accumbal encoding of cue-motivated behavior *via* CB1 receptor activation in the ventral tegmentum. Neuron 73, 360–373. 10.1016/j.neuron.2011.11.018 22284189 PMC3269037

[B44] Oleson E. B. Cheer J. F. (2012). A brain on cannabinoids: the role of dopamine release in reward seeking. Cold Spring Harb. Perspect. Med. 2:a012229. 10.1101/cshperspect.a012229 22908200 PMC3405830

[B46] Parsons L. H. Hurd Y. L. (2015). Endocannabinoid signalling in reward and addiction. Nat. Rev. Neurosci. 16, 579–594. 10.1038/nrn4004 26373473 PMC4652927

[B47] Patel S. Rademacher D. J. Hillard C. J. (2003). Differential regulation of the endocannabinoids anandamide and 2-arachidonylglycerol within the limbic forebrain by dopamine receptor activity. J. Pharmacol. Exp. Ther. 306, 880–888. 10.1124/jpet.103.054270 12808005

[B49] Pfaus J. G. Damsma G. Nomikos G. G. Wenkstern D. G. Blaha C. D. Phillips A. G.. (1990). Sexual behavior enhances central dopamine transmission in the male rat. Brain Res. 530, 345–348. 10.1016/0006-8993(90)91309-5 2176121

[B48] Pfaus J. G. Phillips A. G. (1991). Role of dopamine in anticipatory and consummatory aspects of sexual behavior in the male rat. Behav. Neurosci. 105, 727–743. 10.1037/0735-7044.105.5.727 1840012

[B50] Pickel V. M. Chan J. Kearn C. S. Mackie K. (2006). Targeting dopamine D2 and cannabinoid-1 (CB1) receptors in rat nucleus accumbens. J. Comp. Neurol. 495, 299–313. 10.1002/cne.20881 16440297 PMC1698281

[B51] Rice M. E. Patel J. C. (2015). Somatodendritic dopamine release: recent mechanistic insights. Philos. Trans. R. Soc. Lond. B Biol. Sci. 370:20140185. 10.1098/rstb.2014.0185 26009764 PMC4455754

[B52] Robinson D. L. Heien M. L. Wightman R. M. (2002). Frequency of dopamine concentration transients increases in dorsal and ventral striatum of male rats during introduction of conspecifics. J. Neurosci. 22, 10477–10486. 10.1523/jneurosci.22-23-10477.2002 12451147 PMC6758730

[B53] Rodríguez-Manzo G. (1999a). Blockade of the establishment of the sexual inhibition resulting from sexual exhaustion by the Coolidge effect. Behav. Brain Res. 100, 245–254. 10.1016/s0166-4328(98)00137-5 10212072

[B54] Rodríguez-Manzo G. (1999b). Yohimbine interacts with the dopaminergic system to reverse sexual satiation: further evidence for a role of sexual motivation in sexual exhaustion. Eur. J. Pharmacol. 372, 1–8. 10.1016/s0014-2999(99)00140-5 10374708

[B55] Rodríguez-Manzo G. Canseco-Alba A. (2015). Biphasic effects of anandamide on behavioural responses: emphasis on copulatory behavior. Behav. Pharmacol. 26, 607–615. 10.1097/fbp.0000000000000154 26226145

[B56] Rodríguez-Manzo G. Fernández-Guasti A. (1994). Reversal of sexual exhaustion by serotonergic and noradrenergic agents. Behav. Brain Res. 62, 127–134. 10.1016/0166-4328(94)90019-1 7945962

[B57] Rodríguez-Manzo G. Fernández-Guasti A. (1995). Participation of the central noradrenergic system in the reestablishment of copulatory behavior of sexually exhausted rats by yohimbine, naloxone and 8-OH-DPAT. Brain Res. Bull. 38, 399–404. 10.1016/0361-9230(95)02007-e 8535863

[B58] Rodríguez-Manzo G. Guadarrama-Bazante I. L. Morales-Calderón A. (2011). Recovery from sexual exhaustion-induced copulatory inhibition and drug hypersensitivity follow a same time course: two expressions of a same process? Behav. Brain Res. 217, 253–260. 10.1016/j.bbr.2010.09.014 20875461

[B59] Seif T. Makriyannis A. Kunos G. Bonci A. Hopf F. W. (2011). The endocannabinoid 2-arachidonoylglycerol mediates D1 and D2 receptor cooperative enhancement of rat nucleus accumbens core neuron firing. Neuroscience 193, 21–33. 10.1016/j.neuroscience.2011.07.055 21821098 PMC3579619

[B60] Simerly R. B. Swanson L. W. (1988). Projections of the medial preoptic nucleus: a Phaseolus vulgaris leucoagglutinin anterograde tract-tracing study in the rat. J. Comp. Neurol. 270, 209–242. 10.1002/cne.902700205 3259955

[B61] Stolzenberg D. S. Numan M. (2011). Hypothalamic interaction with the mesolimbic DA system in the control of the maternal and sexual behaviors in rats. Neurosci. Biobehav. Rev. 35, 826–847. 10.1016/j.neubiorev.2010.10.003 20955733

[B62] Stella N. Schweitzer P. Piomelli D. (1997). A second endogenous cannabinoid that modulates long-term potentiation. Nature 388, 773–778. 10.1038/42015 9285589

[B63] Swanson L. W. (1982). The projections of the ventral tegmental area and adjacent regions: a combined fluorescent retrograde tracer and immunofluorescence study in the rat. Brain Res. Bull. 9, 321–353. 10.1016/0361-9230(82)90145-9 6816390

[B64] Tanimura A. Yamazaki M. Hashimotodani Y. Uchigashima M. Kawata S. Abe M.. (2010). The endocannabinoid 2-arachidonoylglycerol produced by diacylglycerol lipase α mediates retrograde suppression of synaptic transmission. Neuron 65, 320–327. 10.1016/j.neuron.2010.01.021 20159446

[B65] Wenkstern D. Pfaus J. G. Fibiger H. C. (1993). Dopamine transmission increases in the nucleus accumbens of male rats during their first exposure to sexually receptive female rats. Brain Res. 618, 41–46. 10.1016/0006-8993(93)90426-n 8402177

[B66] Wenzel J. M. Cheer J. F. (2018). Endocannabinoid regulation of reward and reinforcement through interaction with dopamine and endogenous opioid signaling. Neuropsychopharmacology 43, 103–115. 10.1038/npp.2017.126 28653666 PMC5719091

[B67] Wilson R. I. Nicoll R. A. (2002). Endocannabinoid signaling in the brain. Science 296, 678–682. 10.1126/science.1063545 11976437

[B68] Yang H. de Jong J. W. Tak Y. Peck J. Bateup H. S. Lammel S. (2018). Nucleus accumbens subnuclei regulate motivated behavior *via* direct inhibition and disinhibition of VTA dopamine subpopulations. Neuron 97, 434–449.e4. 10.1016/j.neuron.2017.12.022 29307710 PMC5773387

[B69] Zahm D. S. Cheng A. Y. Lee T. J. Ghobadi C. W. Schwartz Z. M. Geisler S.. (2011). Inputs to the midbrain dopaminergic complex in the rat, with emphasis on extended amygdala-recipient sectors. J. Comp. Neurol. 519, 3159–3188. 10.1002/cne.22670 21618227 PMC3174784

